# Respiratory support strategies in the management of severe, longstanding bronchopulmonary dysplasia

**DOI:** 10.3389/fped.2022.1016204

**Published:** 2022-11-14

**Authors:** J. Wells Logan, Sfurti Nath, Sanket D. Shah, Padma S. Nandula, Mark L. Hudak

**Affiliations:** Department of Pediatrics, College of Medicine - Jacksonville, University of Florida, Jacksonville, FL, United States

**Keywords:** bronchopulmonary dysplasia, respiratory, hypoxemia, hypercarbia, lung resistance

## Abstract

Despite efforts to minimize ventilator-induced lung injury, some preterm infants require positive pressure support after 36 weeks' post-menstrual age. Infants with severe BPD typically experience progressive mismatch of ventilation and perfusion, which manifests as respiratory distress, hypoxemia in room air, hypercarbia, and growth failure. Lung compliance varies, but lung resistance generally increases with prolonged exposure to positive pressure ventilation and other sources of inflammation. Serial lung radiographs reveal a heterogeneous pattern, with areas of both hyperinflation and atelectasis; in extreme cases, macrocystic changes may be noted. Efforts to wean the respiratory support are often unsuccessful, and trials of high frequency ventilation, exogenous corticosteroids, and diuretics are common. The incidence of pulmonary hypertension increases with the severity of BPD, as does the mortality rate. Therefore, periodic screening and efforts to mitigate the risk of PH is fundamental to the management of longstanding BPD. Failure of conventional, lung-protective strategies (e.g., high rate/low tidal-volume and/or high frequency ventilation) warrants consideration of ventilatory strategies individualized to the disease physiology. Non-invasive modes of respiratory support may be successful in infants with mild to moderate BPD phenotypes. However, infants with moderate to severe BPD phenotypes often require invasive respiratory support, and pressure-limited or volume-targeted conventional ventilation may be better suited to the physiology than high-frequency ventilation. The consistent provision of adequate support is fundamental to the management of longstanding BPD and is best achieved with a stepwise increase in ventilator support until comfortable spontaneous respirations are achieved. Adequately supported infants typically experience improvements in both oxygenation and ventilation, which, if sustained, may arrest and generally reverses the course of a potentially lethal lung disease. Care should be individualized to address the most likely pulmonary mechanics, including variable lung compliance, elevated airway resistance, and variable airway obstruction.

## Introduction

The pathophysiology of bronchopulmonary dysplasia (BPD) is complex. While the phenotype evolves principally from the gestational age at birth, the postnatal course and outcome are influenced by the net sum of exposures and events arising before, during, and after delivery. Factors already present at birth, such as intra-uterine growth status, antenatal steroid exposure, and genetic pre-disposition contribute significantly to the evolution of BPD. As the immature lung transitions from a fluid-filled organ appropriate for *in utero* lung development to an air-filled organ appropriate for gas exchange, mechanical and biological forces, exerted over time, modify and damage the structural scaffolding, airways, and pulmonary vascular bed (1). Positive pressure ventilation compresses the interstitium, disrupting the immature collagen network, and preventing normal septation (1). The density of collagen deposition increases with disease severity, the saccules become more disorganized, and V/Q mismatch becomes increasingly apparent (1). Diffusion capacity (relative to lung volume) is decreased among infants with BPD, suggesting that decreased surface area is a mechanism by which gas exchange is impaired (2).

**Figure 1:**
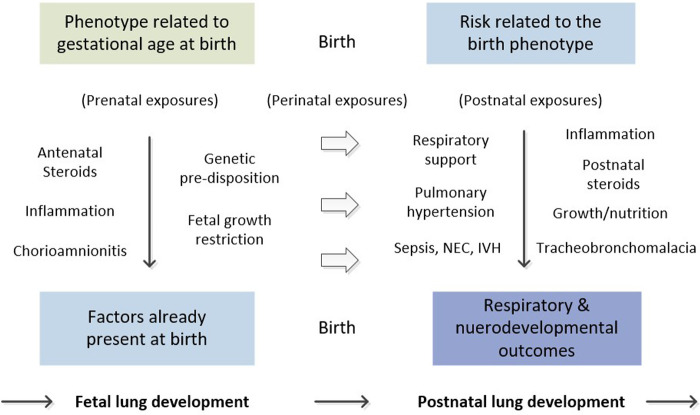
Natural history of bronchopulmonary dysplasia.

Despite efforts to avoid intubation, about one-half of extremely preterm infants require ongoing cycled positive pressure ventilation at age 7 days. Some later fail extubation despite the use of exogenous surfactant and lung-protective strategies, including early extubation, high-frequency ventilation, and fluid restriction (3–5). Unfortunately, the lungs and airways of preterm infants are vulnerable to other postnatal exposures as well (6). For example, early onset sepsis, necrotizing enterocolitis, and other processes that produce a systemic inflammatory response contribute to the evolution of BPD (7). Figure 1 illustrates the complex pathophysiology of BPD, illustrating the interaction of the birth phenotype, which varies significantly, even among infants of the same gestational age, with prenatal, perinatal, and postnatal exposures.

Researchers have identified several phenotypes based on risk factors and the clinical course in the neonatal intensive care unit (NICU) (8). In one study, three distinct disease elements were examined with regard to their contribution to the composite outcome, death before hospital discharge, tracheostomy, or home pulmonary vasodilator therapy. Parenchymal lung disease, pulmonary hypertension (PH), and large airway disease (tracheobronchomalacia) were significantly associated with an increased risk of the composite outcome, and the risk of the primary outcome increased with the number of disease elements documented (8).

## Natural history of BPD: care along the continuum

In the acute phase of respiratory distress syndrome (RDS), the lungs are stiff and non-compliant. Clinical factors such as surfactant administration, fluid balance, and the presence of extrapulmonary shunts, all contribute to changes in compliance. Lung resistance, however, is relatively low in the acute phase of RDS, and typically remains low for days to weeks. Early serial chest radiographs reveal homogeneous, hazy opacities, low lung volumes, and air bronchograms. With the development of less invasive surfactant administration (LISA) and minimally invasive surfactant therapy (MIST) methods, infants requiring surfactant may be stabilized on non-invasive respiratory support, such as nasal CPAP, non-invasive positive pressure ventilation (NIPPV), and rarely, high-flow nasal cannula (HFNC). A lung-protective strategy utilizing short inspiratory times (≤0.35 s), lower tidal volumes (4–6 ml/kg) and higher respiratory rates (30–40 breaths/min) is generally sufficient to achieve respiratory stability in infants who require cycled positive pressure ventilation. This strategy targets low pulmonary resistance and compliance, and improvements are often noted within hours of delivery (9). Extubation success is more likely among infants requiring low concentrations of supplemental oxygen and low levels of non-invasive support, especially those born in high-volume centers with active golden hour protocols (10, 11).

Infants with a more severe BPD phenotype, however, often fail to achieve respiratory stability with non-invasive modes of support. Despite the use of caffeine, rigorous attention to the respiratory support interface, and judicious fluid management, V/Q mismatch progresses with time. Although lung compliance may normalize within days to weeks, lung resistance generally increases with prolonged exposure to positive pressure support. As the lung resistance increases, so does the degree of V/Q mismatch, hypoxemia, and hypercapnia. In time, serial radiographs reveal a more heterogeneous pattern, with areas of both hyperinflation and atelectasis, and, in extreme cases, macrocystic changes may be noted (12). This clinical pattern is sometimes described as “unstable BPD”, and efforts to wean the respiratory support in this phase of care are usually unsuccessful (13). Despite trials of high frequency ventilation, aggressive diuresis, and repeated courses of exogenous corticosteroids, hypoxemia and hypercarbia progress, and growth failure is common.

## Invasive vs. non-invasive respiratory support

Infants requiring mechanical ventilation after 28 days of life are generally considered to have severe, type 1 BPD, even if they are subsequently weaned from mechanical ventilation (14). Infants who require mechanical ventilation at 36 weeks' post-menstrual age are said to have severe, type 2 BPD (14). The vast majority of infants with severe BPD have a significant diffusion defect requiring high mean airway pressures and/or inspired oxygen concentrations to maintain target oxygen saturations and achieve respiratory stability. The diffusion defect varies with the severity of BPD, but airway obstruction and airway malacia are variable (15). Infants with a more severe BPD phenotype are more likely to have airway malacia requiring higher levels of positive end-expiratory pressure, and despite theoretical concerns about the use of bronchodilators some have airway obstruction responsive to bronchodilators (8, 15). In one study, 51% patients had obstructive, 40% had mixed (obstructive and restrictive), and 9% had restrictive phenotypes (15). Bronchodilator response was seen in 74% of infants with an obstructive phenotype, 63% of infants with a mixed phenotype, and 25% of those with a pure restrictive phenotype. Regardless of the clinical phenotype, the treatment strategy should match the disease physiology, and clinicians should strive to individualize the goals of care.

While randomized trials and meta-analyses have documented the benefits of volume-targeted ventilation in the management of acute respiratory distress syndrome (RDS), we await randomized trials to determine the most appropriate mode for management of infants with severe phenotype BPD (16). The provision of adequate respiratory support is imperative, but non-invasive support should be utilized only if it serves to eliminate air hunger, achieve target oxygen saturations on relatively low inspired oxygen, and mitigate the development or evolution of PH. The need for high inspired oxygen concentration or worsening of PH suggests that invasive mechanical ventilation may be necessary. Although there is no universally accepted threshold at which to escalate support, a fraction of inspired oxygen concentration ≥0.65, increased work of breathing, worsening hypercarbia, and/or growth failure suggest that non-invasive support is likely inadequate to arrest or reverse the clinical trajectory. Most worrisome is the development or progression of PH, which is among the most important co-morbidities of BPD, occurring in as many as 50% of infants with severe BPD (17, 18).

## BPD-related pulmonary hypertension

PH contributes significantly to the severity of BPD and, when severe, is associated with a high rate of mortality (19, 20). Unfortunately, the pathophysiology of PH is as complex as that of BPD. Researchers have classified preterm infants into phenotypes based on the onset of PH: early PH (first two weeks after birth), late PH (weeks to months), and chronic/persistent PH (months to years) (20). While the early phenotype may be related to intrinsic/antenatal factors or factors related to intrauterine lung growth and development, the late and chronic phenotypes often reflect the modulating effect of postnatal events and exposures, such as prolonged mechanical ventilation and other biologic/inflammatory exposures (Figure 1).

In the setting of severe parenchymal lung disease, several mechanisms are likely to contribute to the development of PH (17). Air-trapping and hyper-inflation compress or stretch regional pulmonary arteries, thus increasing pulmonary vascular resistance, V/Q mismatch, and hypoxemia. Likewise, regional atelectasis compresses pulmonary blood vessels, limiting pulmonary blood flow and worsening V/Q mismatch and hypoxemia (17). Hypoxemia, in turn, results in acute pulmonary vasoconstriction, and if prolonged, induces pulmonary vascular remodeling characterized by hyperplasia and hypertrophy of vascular smooth muscle. Expert opinion and experience from high-volume centers suggests that optimizing gas exchange is an important component of the treatment plan (17, 18).

Another potentially important mechanism of PH is the presence of extrapulmonary shunts, which can have both short- and long-term effects on the pulmonary vascular bed. Excessive left-to-right shunt (i.e., shunt from the systemic circulation toward the lungs) increases pulmonary vascular resistance and, in time, promotes pulmonary vascular remodeling (17). Unfortunately, the echocardiogram does not easily quantify the magnitude of extra pulmonary shunt, and cardiac catheterization may be needed to characterize the impact of the shunt on pulmonary hemodynamics (18). Over time, large left-to-right shunts progressively impair right ventricular function, which has important management implications. While the provision of adequate respiratory support is the principal treatment of BPD-related PH, pulmonary vasodilators are frequently used as adjuncts, and are best utilized in the context of an inter-disciplinary team that includes pediatric cardiology support (18). Until randomized controlled trials are available, expert opinion and single center case series support a management approach anchored in several concepts: the provision of respiratory support sufficient to minimize work of breathing, maintenance of oxygen saturations within a safe target range, and the employment of pulmonary vasodilators on a selective basis (18).

## Clinical recognition of longstanding BPD: when to consider an alternative approach?

There are currently no multicenter randomized controlled trials addressing the most appropriate timing or alternative approaches to the lung protective strategies used to prevent BPD. Consensus among collaborating BPD centers has highlighted the importance of interdisciplinary teams, especially with regard to optimizing respiratory support and nutrition, and mitigating the development or evolution of PH, but also to improve the quality and consistency of care (21). Clinical indicators for an alternative respiratory strategy includes: sustained respiratory distress, recurrent cyanotic or bradycardic episodes, intolerance of physical therapy and handling, poor growth, and repeated courses of systemic corticosteroids—without benefit (21). Unfortunately, no single respiratory support strategy has been shown to improve the outcomes of infants with severe, longstanding BPD. For now, the best available evidence derives from consensus recommendations and small, single center series with extensive experience and favorable outcomes (21–23).

In a study of ventilator-dependent infants who underwent tracheostomy in Denver, CO, the authors compared survival from two eras of care that employed two very different respiratory support strategies (23). In the first era, clinicians used a standard approach. In the second, an individualized care program utilized patient-specific ventilator settings, optimized lung volumes, and higher positive end-expiratory pressure (PEEP) for infants with airway malacia. The care protocol included a focus on meticulous clearance of airway secretions, universal periodic screening for PH, and prompt treatment of factors contributing to parenchymal lung injury. Survival increased from 50% in the first era to 85% in the second (23).

In a study of 71 BPD patients in Columbus, OH, infants with longstanding BPD were referred at a median PMA of 47 weeks (IQR, 42, 53) and had a median respiratory severity score (RSS) of 8.1 (IQR 4.5, 11.0) on admission (22). A dedicated multi-disciplinary team used a patient-specific ventilator strategy tailored to the most likely physiology, with an emphasis on achieving a pro-growth, pro-development state. Despite initiating this management late in the course, when infants had already developed severe disease, over 92% of patients survived to hospital discharge with improvement in comorbidities (22).

Increasing oxygen requirements, persistent hypercarbia, multiple failed attempts at extubation, and recurrent courses of postnatal steroids are all clinical markers of severe BPD. Therefore, we recommend consideration of an alternative respiratory support strategy when acute, lung-protective strategies have failed and adjunctive therapies have been exhausted, including the treatment of persistent, hemodynamically significant PDA. Objective signs of failure of lung-protective strategies include severe V/Q mismatch (hypoxemia and hypercapnia), a heterogeneous pattern on serial radiographs, and failure to benefit from high frequency ventilation, diuretics, and corticosteroids. Every effort should be made to use a lung protective strategy, and an alternative approach should be considered only when these efforts have failed.

Premature utilization of a modified/chronic approach may lead to unintentional lung injury, further complicating respiratory management. One such example is the presence of a persistent PDA. The clinical markers of a hemodynamically significant PDA include left atrial enlargement, left ventricular enlargement (suggesting over-circulation), and a rising creatinine, with or without acidosis. This scenario is common, but potentially reversible. Corrective interventions should be explored before considering an alternative respiratory support strategy. In recent years, non-invasive techniques for PDA closure have been introduced with very promising results (24–26). Potentially modifiable and/or acute respiratory maladies should be treated before moving to a chronic phase treatment strategy.

The radiographic pattern can be helpful in determining if or when to modify the management strategy. Some centers utilize high-resolution CT imaging for phenotyping, whereas others use a combination of radiographs and clinical experience (8, 23). The radiographic pattern is generally predictable, with regions of scattered, multi-focal atelectasis superimposed on regions of hyperinflation. This radiographic pattern suggests that some areas of the lung are engaged in respiration, while others are not. As the severity of BPD evolves, so too does the radiographic pattern, and in extreme cases, small and large pneumatoceles can signify a more severe BPD phenotype. In time, the mechanics of respiration evolve, and infants with severe disease will have regional variations in lung compliance and resistance within the lung compartment.

Bedside measures of lung function are generally unreliable, providing only a snapshot of the compliance and resistance of the respiratory system. Nonetheless, the time constant for acute lung diseases (e.g., RDS) is relatively short. This contrasts with the respiratory mechanics of longstanding BPD, where regional differences in lung compliance and resistance are common and where the overall resistance is much higher than that of infants with RDS. Stated differently, in severe, longstanding BPD some regions of the lung compartment have high resistance correlating with longer time constants (the slow compartment), while other regions of the lung compartment have low resistance and shorter time constants (the fast compartment). Theoretically, the slow compartment dominates the physiology of infants with severe, longstanding BPD, so the respiratory support strategy should target the slow compartment.

## An alternative respiratory support strategy for infants with severe, longstanding BPD

Use of an acute, lung-protective strategy (high rate, low tidal volume) in infants with severe longstanding BPD typically results in failure to ventilate a large portion of the lungs during the respiratory cycle, which results in worsening of V/Q mismatch, air hunger, and air-trapping (12). Air-trapping, in turn, results in dynamic collapse of regional airways, which by compressing the pulmonary vascular bed, only worsens V/Q mismatch (diffusion defect) and its downstream effects.

Because high lung resistance and airway obstruction dominate the physiology, the respiratory strategy must target the disease physiology—variable lung compliance, high lung resistance, and airway obstruction for the vast majority of patients. In order to adequately engage the slow compartment (high lung resistance), longer inspiratory times and larger tidal volumes are required to improve V/Q matching (13). In extreme cases, inspiratory times as long as 0.6–0.8 s and tidal volumes as high as 10–15 ml/kg may be needed. Elastic recoil is significantly impaired in severe BPD, which manifests clinically as airway obstruction (15). Therefore, lower ventilator rates are needed to facilitate passive emptying of the lungs. Ventilator rates as low as 12–16 breaths/min and I:E ratios ≥1 to 3.5 may be needed to facilitate carbon dioxide clearance. Over 70% of infants with severe BPD demonstrate airway obstruction responsive to bronchodilators, including infants with a mixed obstructive/restrictive phenotype (15). As such, the use of inhaled beta-agonists can facilitate carbon dioxide clearance, and ventilator graphics can be used to estimate the end of each exhalation cycle (15, 27).

Despite consensus recommendations from collaborating centers, significant variations in care have been documented (21). In a recent publication from the Children's Hospital Neonatal Consortium (CHNC), researchers found significant differences in ventilator modes and practices; 51% were treated with volume-control or volume guarantee, 43% with pressure-control, and 6% with neurally adjusted ventilatory assist (NAVA) (28). The use of non-invasive modes was equally variable; 41% were treated with high-flow nasal cannula (HFNC), 28% with low-flow nasal cannula (LFNC), 26% with continuous positive airway pressure (CPAP), and 5% with other modes (28). Likewise, in a study of 700 infants with BPD, published in 2015, marked differences were noted in the use of mechanical ventilation, diuretics, inhaled corticosteroids, and inhaled beta-agonists (29). The timing and prevalence of tracheostomy insertion are also variable, especially among infants with BPD-related PH and those born small for gestational age (SGA) (30, 31).

Consistent care and enhanced team communication are marks of exemplary BPD care that translate into improvements in respiratory outcomes and survival (23, 32, 33). Data on long-term respiratory outcomes suggests that infants with severe BPD are at greater risk for airway obstruction and hospital re-admission extending into adolescence and adulthood (27, 34). However, data from population based studies is sparse and lacks the granularity needed to correlate clinical strategies with outcomes (35–37). Nonetheless, use of a ventilatory approach that is tailored to the most likely physiology, together with the use of inter-disciplinary teams and guidelines has been shown to improve both care and outcomes (21, 33).

## Goals of respiratory management for severe, longstanding BPD

The goals of management for severe, longstanding BPD are different from those related to the prevention of BPD. Whereas lung-protective strategies are designed to minimize exposure to positive pressure ventilation, the goals of management for severe longstanding BPD center around the three major aims: (1) providing ventilatory support sufficient to achieve comfortable work of breathing, (2) minimizing exposures that interfere with lung growth, and (3) furnishing an environment that optimizes both lung and brain development (12, 38). Performing a comprehensive physical exam is fundamental to the first goal, as it is important to match the respiratory support strategy with the infant's respiratory and metabolic needs. Specifically, the respiratory support settings should be increased, incrementally, until the infant has achieved comfortable respirations, both at rest and during age-appropriate cares and activities.

Conventional ventilation modes (pressure or volume regulated) are recommended for infants who require positive pressure ventilation. In general, higher peak inspiratory pressures (and/or tidal volumes) are needed to alleviate air-hunger and respiratory distress. Once the air hunger/distress has been alleviated, the respiratory support should be maintained at a level sufficient to maintain stable oxygen saturations, comfortable work of breathing, and adequate growth. Increased work of breathing interferes with lung growth by expending nutritional reserves that promote growth. Once the patient is adequately supported, the oxygen saturation has been stabilized within an accepted target range (e.g., ≥94%), and linear growth has been documented, the FiO2 can be reduced in small increments. While great care should be exercised to avoid wide swings in oxygen saturation, the ability to reduce the FiO2 sequentially is an important marker of clinical improvement.

Care should be individualized and modified in consideration of disease severity. Decreasing the level of support should be avoided until substantial gains in respiratory reserve and linear growth have been achieved (22, 38). Premature reduction of the level of support could result in respiratory failure, growth failure, and/or pulmonary hypertension. Specifically, efforts to reduce the respiratory support should be avoided until the oxygen requirement is consistently less than 40% or until consistent hypocapnia (e.g., PCO2 < 35) mandates a reduction in tidal ventilation. Hypercarbia is common among infants with BPD, especially among those with severe phenotype BPD. Carbon dioxide retention is better assessed with serial (weekly) bicarbonate levels than with serial blood gases, so once the patient has reached a chronic/stable phase of BPD, the frequency of blood gases can be reduced or reserved for acute clinical deteriorations (12).

Another important marker of clinical improvement is the infant's tolerance for age-appropriate developmental challenges (e.g., age-appropriate play, upright positioning, and social interactions with nurses and parents). Once the patient has achieved clinical stability, demonstrated by tolerance for age-appropriate activities, and has reached a sustained pro-growth state, then a cautious, stepwise reduction of the respiratory support apparatus should be considered (13). For infants with severe, longstanding BPD, this may not occur until weeks to months after the initial stabilization. The most appropriate interval for weaning the support apparatus depends on the phenotype, disease severity, the level of stability achieved, and progress toward goals.

## Conclusion

Until results of adequately powered randomized trials are available, the respiratory support strategy of infants with severe, longstanding BPD should target the most likely physiology. We advocate an approach taken by centers documenting improvements in both lung function and survival (15, 21–23). Severe, established BPD is characterized by high respiratory system resistance, which correlates with longer time constants and variable airway obstruction. For infants who require mechanical ventilation, longer inspiratory times and higher tidal volumes are needed to overcome V/Q mismatch (12). Likewise, infants with severe, longstanding BPD typically have impaired elastic recoil and airway obstruction, and generally benefit from lower ventilator rates to facilitate passive emptying of the lungs. Continuous provision of adequate respiratory support anchors the approach to both the prevention and treatment of BPD-related PH. Expert opinion supports the maintenance of oxygen saturations within a safe target range and the use of pulmonary vasodilators as an adjunctive PH therapy in collaboration with a pediatric cardiologist (26). Finally, an inter-disciplinary team approach is key to tracking progress toward respiratory goals, cardiac function and hemodynamics, somatic, linear, and end-organ growth, and overall progress toward the goal of successful discharge (21).
